# Grip strength from midlife as an indicator of later-life brain health and cognition: evidence from a British birth cohort

**DOI:** 10.1186/s12877-021-02411-7

**Published:** 2021-08-31

**Authors:** Quentin Dercon, Jennifer M. Nicholas, Sarah-Naomi James, Jonathan M. Schott, Marcus Richards

**Affiliations:** 1 MRC Cognition and Brain Sciences Unit, University of Cambridge, Cambridge, United Kingdom; 2 Department of Medical Statistics, London School of Hygiene & Tropical Medicine, London, United Kingdom; 3 MRC Unit for Lifelong Health and Ageing at UCL, University College London, London, United Kingdom; 4 Dementia Research Centre, UCL Queen Square Institute of Neurology, University College London, London, United Kingdom

**Keywords:** Grip strength, Physical function, Brain volume, White matter hyperintensity volume, Nonverbal reasoning, Cognitive ageing

## Abstract

**Background:**

Grip strength is an indicator of physical function with potential predictive value for health in ageing populations. We assessed whether trends in grip strength from midlife predicted later-life brain health and cognition.

**Methods:**

446 participants in an ongoing British birth cohort study, the National Survey of Health and Development (NSHD), had their maximum grip strength measured at ages 53, 60–64, and 69, and subsequently underwent neuroimaging as part of a neuroscience sub-study, referred to as “Insight 46”, at age 69–71. A group-based trajectory model identified latent groups of individuals in the whole NSHD cohort with below- or above-average grip strength over time, plus a reference group. Group assignment, plus standardised grip strength levels and change from midlife were each related to measures of whole-brain volume (WBV) and white matter hyperintensity volume (WMHV), plus several cognitive tests. Models were adjusted for sex, body size, head size (where appropriate), sociodemographics, and behavioural and vascular risk factors.

**Results:**

Lower grip strength from midlife was associated with smaller WBV and lower matrix reasoning scores at age 69–71, with findings consistent between analysis of individual time points and analysis of trajectory groups. There was little evidence of an association between grip strength and other cognitive test scores. Although greater declines in grip strength showed a weak association with higher WMHV at age 69–71, trends in the opposite direction were seen at individual time points with higher grip strength at ages 60–64, and 69 associated with higher WMHV.

**Conclusions:**

This study provides preliminary evidence that maximum grip strength may have value in predicting brain health. Future work should assess to what extent age-related declines in grip strength from midlife reflect concurrent changes in brain structure.

**Supplementary Information:**

The online version contains supplementary material available at 10.1186/s12877-021-02411-7.

## Background

Grip strength is an objective measure of upper limb strength and an indicator of overall physical function [1]. It changes with age, reaching a plateau around age thirty before beginning to decline after age fifty [2]. Men generally have higher maximum grip strength than women after controlling for body size [3], with a delayed but steeper decline later in life [2]. Growing evidence suggests that levels and changes in grip strength with age may reflect the capacity to specify and coordinate motor commands, and may be sensitive to subtle changes in brain health [4], leading some to argue that grip strength may have prognostic value in ageing populations [5, 6]. For example, grip strength has been linked to multiple negative outcomes including lower later-life physical capability [1], higher rates of later-life disability [7] and mortality [8, 9], and cognitive impairment [10], even when measured decades earlier in midlife [7, 8] (around 45 to 65 years of age [11]).

Studies investigating the extent to which grip strength is associated with cognitive function have mixed results. Systematic reviews have indicated some evidence for positive cross-sectional associations between grip strength and cognitive function, and for longitudinal associations between their rates of change [12], but only limited evidence for associations between grip strength and subsequent cognitive decline [13]. Although these discrepancies are partly due to heterogeneity in cohorts and measures, cognitive tests may be insensitive to subtle changes in neurological function. To overcome this issue, recent studies have examined neuroimaging-derived indices, such as whole-brain atrophy and white matter hyperintensity volume (WMHV), an index of presumed small vessel disease [14]. While declines in whole-brain volume (WBV) and increases in WMHV occur in healthy ageing [15, 16], both have been linked to negative health outcomes including dementia and cognitive decline [17, 18]. There is some cross-sectional evidence linking lower grip strength to increased WMHV [19] and lower WBV [20], though other studies have reported null findings [21].

This study builds on these findings using the National Survey of Health and Development (NSHD; the British 1946 birth cohort). Following an original sample of 5,362 men and women born in a single week in March 1946, the NSHD contains a wealth of life-course data including physical and cognitive measures [22]. In addition, 502 participants were selected for its neuroscience sub-study, Insight 46, which incorporated cognitive testing with MRI and PET neuroimaging at age 69-71 [23]. The present study assessed relationships between grip strength from midlife, and brain health and cognition at 69–71. We speculated that physical performance is influenced by concurrent neurological health, and hypothesised that below-average trends in grip strength from midlife predict lower WBV and increased global WMHV, and below-average cognitive performance.

## Methods

### Participants

A total of 502 individuals from the NSHD were recruited for the Insight 46 neuroscience sub-study at age 69–71 [24]. Of these, 446 participants (*n* = 218 females; 48.9 %) had complete MRI data (WBV and global WMHV) plus complete data for at least one of the whole-cohort nurse visits at age 53 [25], 60–64 [26] or 69 [22] (see Figure S1 for an inclusion flow-chart).

### Measures

#### Grip strength at 53, 60–64, and 69

Maximal grip strength was measured at each of the three nurse visits using a calibrated handgrip dynamometer, and defined as the maximum of four measures (two for each hand) [27]. Given known sex differences, including in this cohort [3], raw maximum grip strength (in kg) was converted to a within-sex z-score (mean subtracted and divided by the standard deviation (SD)), based on the measures from the whole study sample (*n* = 2,850 at 53; *n* = 2,069 at 60–64; *n* = 2,103 at 69).

#### Cognitive & neuroimaging outcomes at 69–71

Cognitive tests were administered as part of Insight 46 [23]. These included tests of associative memory (FNAME-12 [28]), episodic memory (Logical Memory IIa delayed recall [29]), psychomotor speed and executive function (DSST [30]), and cognitive impairment (MMSE [31]). Scores for each test were converted to z-scores based on all 502 Insight 46 participants [32], and the z-scores for each participant averaged to give a version of the Preclinical Alzheimer Cognitive Composite (PACC [33]). A test of matrix reasoning was also administered, which assesses nonverbal reasoning ability [34].

The neuroimaging metrics of interest, global WMHV (mL) and WBV (cm^3^), were obtained from a single 60 min scan using a Biograph mMR 3T MRI/PET scanner (Siemens Healthcare, Erlangen, Germany). Scans were completed during the same visit as the cognitive tests for all but 58 of the 446 participants. WBV was derived from 3D T1-weighted MRI using an automated segmentation procedure [35] followed by manual checks and edits. Global WMHV, including subcortical grey matter but not infratentorial regions, was derived from multimodal MRI using an automated segmentation algorithm, BaMoS [36].

#### Covariates

Covariates were sex; age, height (in cm) and weight (in kg) at each nurse visit; adult Registrar General’s socioeconomic position (SEP) at 53 grouped into non-manual and manual; and education level defined as the highest educational qualification achieved by 26, categorised into no formal qualifications, secondary school leavers exams, or any degree.

Given known links between the neuroimaging metrics in this study and vascular risk factors [37], cardiovascular risk measures were included by deriving office-based Framingham Heart Survey cardiovascular disease (FHS-CVD) risk scores at each nurse visit [38]. Physical activity at each nurse visit was defined as the number of times participants reported taking part in activities requiring physical exertion in the prior four weeks, categorised into inactive (none), moderately active (1–4 times), or highly active (5 + times) [39]. This measure has been shown to be strongly associated with grip strength in this cohort [40]. In addition, a binary variable was derived to indicate whether participants showed evidence of a cognitive or neurological condition at age 69–71, as assessed by a structured clinical interview [23].

Additional covariates specific to neuroimaging were age at the scan and total intracranial volume (TIV) derived using an automated procedure from the SPM12 package for MatLab [41]. Additional covariates specific to cognitive outcomes were age at the Insight 46 visit, and childhood cognition measured as general cognitive ability at age 15 (*n* = 414), or at 11 (*n* = 21) or 8 (*n* = 11) if missing [42].

### Statistical Analyses

Prior to statistical analyses, continuous variables not already z-scored (age, height, weight, and FHS-CVD risk score at each nurse visit) were mean-centred on the analytical sample. For height and weight, this was done separately in males and females. All statistical analyses were conducted using Stata 16.1 (StataCorp, College Station, TX).

#### Associations between grip strength levels and changes from age 53, with neuroimaging and cognition at age 69–71

Multivariable linear regression models were used to quantify associations between grip strength at 53, 60–64, and 69 and PACC, matrix reasoning, and WBV. Due to distributional skew, bias-corrected and accelerated (BCa) bootstrapping with 2,000 replications was used to obtain valid 95 % confidence intervals (CIs) for associations with matrix reasoning, while gamma family generalised linear models (GLMs) with log link function were used to quantify corresponding associations with WMHV, in line with previous work in this cohort [32, 43]. The same approaches were used to relate each outcome to grip strength score change between 53 and 69. Since the z-scores were calculated at each study wave, a change of 0 between age 53 and 69 indicates an average level of decline, and positive values indicate above-average decline. 

All models were adjusted for sex, age at the nurse visit(s) and the Insight 46 scan or visit, height, weight, sociodemographic factors, vascular and health risk factors, and presence of cognitive or neurological impairment at 69–71. In the models for change in grip strength, life-course covariates were from the baseline measure (age 53). All models with cognitive tests as the outcome also adjusted for childhood cognition; models for neuroimaging metrics also adjusted for TIV; and models for WMHV also adjusted for weight squared (in addition to weight) given evidence for a quadratic effect of weight from residual plots. A sex by grip strength interaction was also tested, with this term included if there was evidence for such an effect at *p* < 0.1; coefficients and CIs were then presented for each sex. Model fit was assessed for all models via residual plots.

#### Group-based trajectory modelling

Group-based trajectory modelling (GBTM) was used in an exploratory manner to identify groups of individuals following similar grip strength trajectories over time. GBTM identifies latent groups of individuals based on their patterns of responses, and models each group separately [44]. Notably, as GBTM can handle missing data, all individuals in the whole NSHD sample with at least one grip strength measure at age 53, 60–64 or 69 (*n* = 3078) were included in the model. Posterior probabilities of group membership were calculated for each individual for each group; the maximum of these was used to classify each participant into their most likely group. Models with increasing numbers of groups were fitted, and in line with recommendations [45], the optimal number of groups was identified using the Bayesian Information Criterion (BIC) and the mean posterior probability of membership in each group among those assigned that group (a metric of classification ambiguity [46]). Group assignment was then related to each of the outcomes, with the largest group taken as the reference. These models adjusted for the same covariates as those relating change in grip strength from midlife to the outcomes, except that they excluded age at the time of the nurse visits (as this was included in the GBTM). See Supplementary Data Sec. 1 for further details.

## Results

**Fig. 1:**
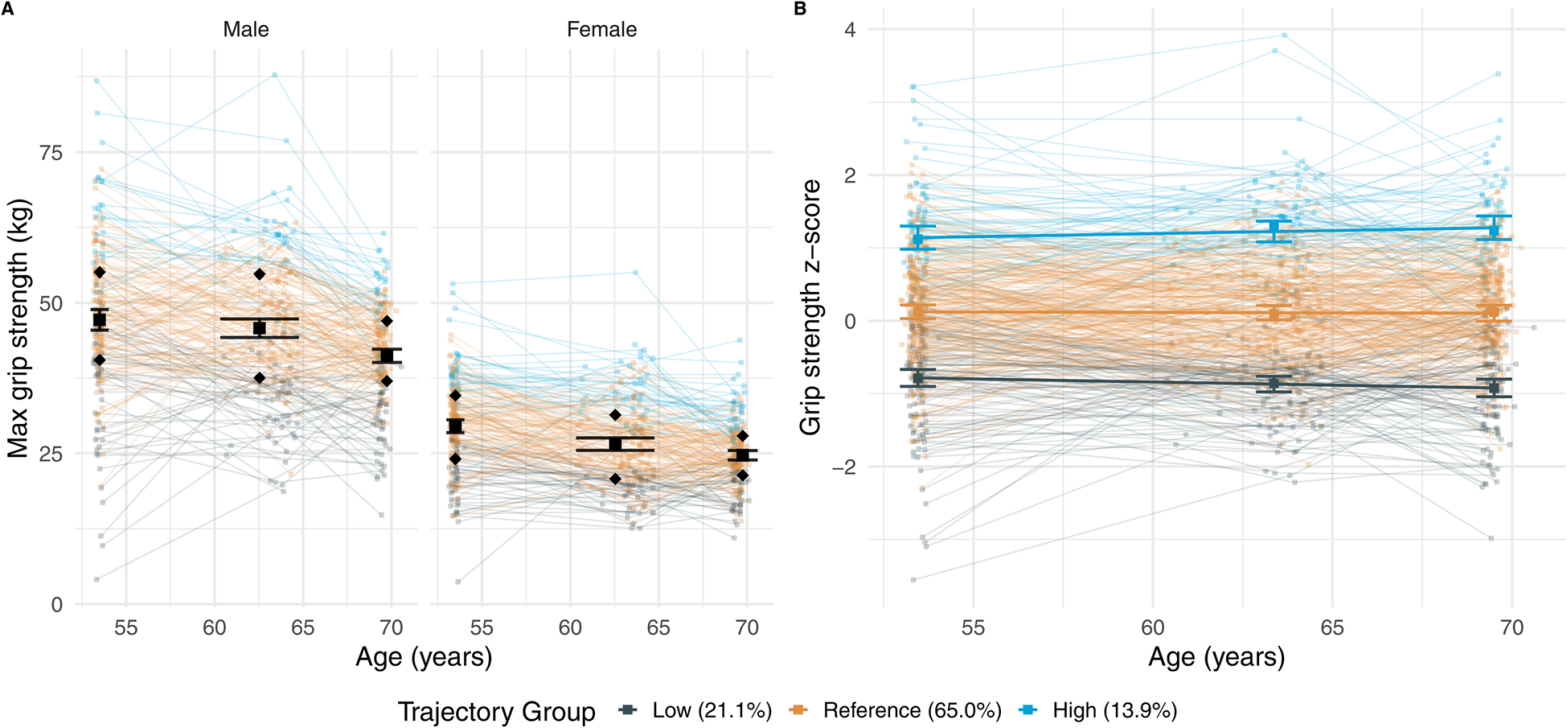
Trajectories of grip strength from age 53 to 69 in included participants (*n* = 446). **A** Individual maximum grip strength trajectories in all included Insight 46 participants, stratified by sex, and coloured by their assigned group from the GBTM based on a total of 3,078 NSHD participants (*n* = 7019 individual observations). Mean grip strength ± standard error (SE) error bars plus lower and upper quartiles (shown as diamonds) are shown for the included Insight 46 participants for each of the three study waves (age 53, 60–64, and 69). **B** Grip strength z-score trajectories for all included Insight 46 participants, coloured by their assigned trajectory group. Bold lines correspond to the estimated trajectory for each of the groups derived from the GBTM, with error bars showing 95 % CIs at the mean age of each study wave, and the bold squares are the mean grip strength z-score for each group at each study wave; note that the mean z-scores, estimated mean trajectories, and error-bars are based on all NSHD participants included in the model (*n* = 3078)

In the 446 participants, maximum grip strength fell from an average of 47.2 kg (SD = 12.7; *n* = 211) at age 53, to an average of 41.2 kg (SD = 8.3; *n* = 217) at age 69 in men, and from an average of 29.5 kg (SD = 7.9; *n* = 205) at age 53 to an average of 24.7 kg (SD = 5.5; *n* = 205) at age 69 in women (Fig. 1A). The average decline from age 53 to 69 was 5.6 kg (SD = 11.8; *n* = 200) in men, and 5.0 kg (SD = 7.6; *n* = 193) in women. Summary statistics for grip strength and all covariates are presented in Table 1 (by sex where relevant), alongside those for all others in the whole NSHD sample with complete records at each time point. As the raw grip strength measures were converted to within-sex z-scores, all results are given as the effect of a 1 SD increase in maximum grip strength on each of the outcomes.

**Table 1:** Participant characteristics comparing included Insight 46 participants and all other NSHD participants with complete records

		Age 53 (omitted)	Age 53 (included)	*p*-value	Age 60-64 (omitted)	Age 60-64 (included)	*p*-value	Age 69 (omitted)	Age 69 (included)	*p*-value
No. with complete records	**M:** **F:**	962 (49.0) 1000 (51.0)	211 (50.7) 205 (49.3)	0.53	599 (48.0) 649 (52.0)	208 (51.2) 198 (48.8)	0.25	681 (48.5) 722 (51.5)	217 (51.4) 205 (48.6)	0.30
Age, years	**M:** **F:**	53.5 (0.2) 53.5 (0.2)	53.4 (0.2) 53.4 (0.2)	0.12 0.01	63.2 (1.2) 63.4 (1.1)	63.4 (1.0) 63.3 (1.1)	0.10 0.54	69.5 (0.2) 69.5 (0.2)	69.5 (0.2) 69.5 (0.2)	0.02 0.09
***Physical performance***										
Max grip strength (kg)	**M:** **F:**	47.8 (12.0) 27.5 (7.9)	47.2 (12.7) 29.5 (7.7)	0.62 <0.001	44.5 (11.7) 26.2 (7.4)	45.8 (11.3) 26.5 (7.4)	0.15 0.60	39.9 (8.6) 24.0 (5.9)	41.2 (8.3) 24.7 (5.5)	0.04 0.12
***Body size***										
Height (cm)	**M:** **F:**	174.6 (6.5) 161.6 (5.9)	175.1 (6.1) 162.2 (5.6)	0.25 0.14	174.7 (6.6) 161.7 (5.9)	175.5 (5.9) 162.2 (5.4)	0.13 0.37	173.8 (6.4) 160.4 (5.9)	174.1 (6.2) 161.1 (5.5)	0.56 0.12
Weight (kg)	**M:** **F:**	83.4 (13.8) 71.4 (14.2)	83.1 (11.3) 69.9 (12.2)	0.77 0.17	85.6 (13.7) 73.5 (15.4)	84.7 (11.9) 71.7 (12.8)	0.42 0.14	83.8 (12.0) 70.3 (13.1)	83.8 (12.0) 70.3 (13.1)	0.12 0.02
***Sociodemographics; N (%)***										
Adult SEP	Manual	708 (36.1)	64 (15.4)	<0.001	398 (31.9)	67 (16.5)	<0.001	451 (31.1)	62 (14.7)	<0.001
	Non-manual	1254 (63.9)	352 (84.6)		850 (68.1)	339 (83.5)		952 (67.9)	360 (85.3)	
Education level (at 26)	No formal	1152 (58.7)	135 (32.4)		668 (53.5)	127 (31.3)	<0.001	782 (55.7)	133 (31.5)	
	School exams	671 (34.2)	210 (50.5)		474 (38.0)	207 (51.0)		513 (36.6)	218 (51.7)	
	Degree+	139 (7.1)	71 (17.1)		106 (8.5)	72 (17.7)		108 (7.7)	71 (16.8)	
***Behavioural and health risk factors***										
Physical activity (last 4 weeks); *N* (%)	None	1005 (51.2)	130 (31.2)	<0.001	827 (66.3)	208 (51.2)		863 (61.5)	185 (43.8)	<0.001
	1-4 times	331 (16.9)	93 (22.4)		156 (12.5)	79 (19.5)		183 (13.0)	62 (14.7)	
	5+ times	626 (31.9)	193 (46.4)		265 (21.2)	119 (29.3)		357 (25.5)	175 (41.5)	
FHS-CVD risk score		11.1 (3.2)	9.8 (2.7)	<0.001	14.3 (3.2)	13.5 (2.9)	<0.001	15.7 (3.2)	15.1 (3.0)	<0.001
***Baseline cognition***										
Childhood cognitive ability (z-score)		0.1 (0.9)	0.6 (0.8)	<0.001	0.1 (0.9)	0.6 (0.8)	<0.001	0.1 (0.9)	0.6 (0.8)	<0.001

* Occupation at age 53 or imputed from earlier in life if missing

† Office-based FHS-CVD risk scores were calculated for each participant as per the supplementary tables in D’Agostino *et al.* (2008) based on their age, sex, body mass index (BMI), systolic blood pressure (SBP; medicated or unmedicated), diabetes status, and smoking status

‡ General cognitive ability was tested at age 15, age 11, and/or age 8, and standardised based on all study participants at each age

‘Omitted’ refers to those in the whole NSHD with complete records at each study wave who were not included in the analysis of cognitive and neuroimaging outcomes; ‘included’ refers to those in the present study (total *n* = 446). Continuous measures are given as mean (SD) to 1 decimal place (d.p.), and categorical variables are given as number (%). *P*-values are from chi-squared tests for categorical variables, and two-tailed t-tests for continuous variables

### Associations between grip strength levels and changes from age 53, and neuroimaging and cognition at age 69–71

**Fig. 2:**
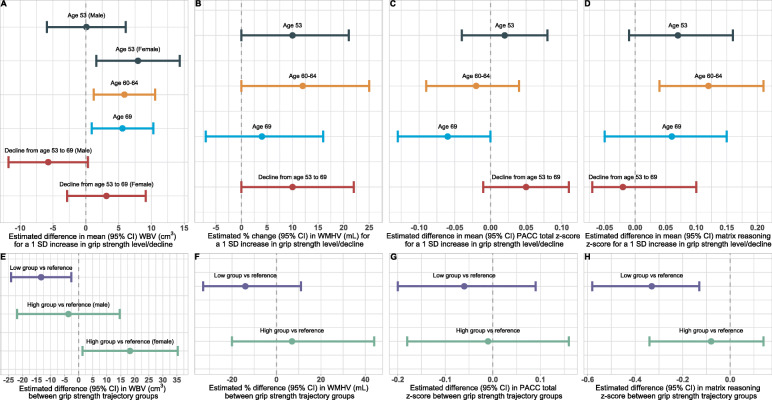
Associations between grip strength from midlife and brain health and cognitive measures at age 69–71. Estimated change (95 % CI) in each of the brain health and cognitive outcomes at age 69–71 for a one z-score increase in levels or declines in maximum grip strength from age 53 to 69 (**A**-**D**) or for membership of the low or high grip strength trajectory groups (compared to reference individuals) (**E**-**H**), derived from fully adjusted linear regression models or GLMs

Higher grip strength at age 53, 60–64, and 69 was associated with increased WBV adjusting for sex, body size, TIV, sociodemographics, and behavioural and health risk factors (Table 2; Fig. 2A). At age 53, there was weak evidence for a sex by grip strength interaction (*p* = 0.075): a 1 standard deviation (SD) increase in maximum grip strength in females was associated with 7.95cm^3^ increased WBV up to 17 years later (95 % CI=(1.61, 14.30)) but little effect was seen in males (0.10cm^3^ increase, 95 % CI=(-5.91, 6.10)).

**Table 2:** Associations between grip strength levels/changes from midlife and brain health and cognitive measures at 69–71

		Max grip at 53 (*n* = 416)	Max grip at 60-64 (*n* = 406)	Max grip at 69 (*n* = 422)	Change in grip from 53 to 69 (*n* = 393)
***Brain health measures***					
WBV (cm^3^)	**M**:	0.10 (-5.91, 6.10)	5.89* (1.22, 10.56)	5.59* (0.90, 10.29)	-5.72 (-11.76, 0.32)
	**F**:	7.95* (1.61, 14.30)			3.15 (-2.84, 9.14)
Global WMHV (mL)		1.10 (1.00, 1.21)	1.12 (1.00, 1.25)	1.04 (0.93, 1.16)	1.10 (1.00, 1.22)
***Cognitive measures***					
PACC (z-score)		0.02 (-0.04, 0.08)	-0.02 (-0.09, 0.04)	-0.06 (-0.13, 0.00)	0.05 (-0.01, 0.11)
Matrix reasoning (z-score)‡		0.07 (-0.01, 0.16)	0.12† (0.04, 0.21)	0.06 (-0.05, 0.15)	-0.02 (-0.07, 0.10)

* *p* < 0.05

† BCa bootstrap 95% CI does not contain 0

‡ 95% CIs obtained from BCa bootstrap with 2,000 replicates

Coefficients (95% CIs) are derived from multivariable linear regression models or GLMs and represent the effect of a one z-score increase in grip strength on mean WBV (cm^3^), mean z-score (cognitive measures) or the multiplicative effect for WMHV (as this is the exponentiated coefficient from the GLM). Z-scores for maximum grip strength were calculated based on the whole NSHD cohort at each nurse visit; z-scores for cognitive tests were calculated based on all 502 Insight 46 participants. Models for levels included those with complete records at that age, models for changes included those with complete records at both age 53 and 69; all 446 participants were hence included in analyses for at least one time point. Decline was calculated as initial grip strength z-score minus final z-score, so coefficients represent the estimated change in the outcome measure (or multiplier for WMHV) for a unit decline in z-score. All models were adjusted for sex, age at scan/visit and nurse visit, body size (weight and height at nurse visit), sociodemographic factors (adult SEP and education level), and sociodemographic, behavioural and health risk factors (physical activity and vascular risk score at nurse visit), plus a binary indicator of cognitive or neurological impairment at 69-71. Models for brain measures additionally controlled for TIV; models for cognitive measures additionally controlled for childhood cognitive ability; and models for WMHV at 53 were additionally adjusted for weight squared. Values given to 2 d.p.

At later study waves, there was limited evidence for a sex difference, with 1 SD increases in maximum grip strength at age 60–64 and 69 predicting an estimated 5.89cm^3^ (95 % CI=(1.56, 10.82)) and 5.59cm^3^ (95 % CI=(0.90, 10.29)) higher WBV at age 69–71 respectively. In line with these results, there was also a trend suggesting that faster decline in grip strength between 53 and 69 was associated with lower WBV in males (*p* = 0.029 for sex by grip strength interaction) but the trend was in the opposite direction for females, albeit even weaker (Table 2).

The associations of grip strength with WMHV were less consistent (Fig. 2B). Unexpectedly, there was some evidence that increased grip strength at age 53 and 60–64 (but not 69) was associated with 10–12 % higher WMHV at age 69–71 (GLM coefficients 1.10 and 1.12 respectively) in adjusted models. In contrast, 1 SD greater decline in maximum grip strength between 53 and 69 was associated with an 10 % increase in WMHV at age 69–71 (coefficient = 1.10; 95 % CI=(1.00, 1.22)) after adjustment. However, the statistical evidence for these effects was fairly weak (Table 2).

For cognitive outcomes, there was a consistent trend that higher grip strength was associated with higher scores in matrix reasoning at 69–71 adjusting for sex, body size, childhood cognition, sociodemographics, and behavioural and health risk factors (Fig. 2D). The strongest evidence for this was at age 60–64 where a 1 SD increase in maximum grip strength was associated with a 0.12 increase in matrix reasoning score (BCa bootstrapped 95 % CI=(0.04, 0.21)); the evidence for effects at age 53 (coefficient = 0.07, 95 % CI=(-0.01, 0.16)) and age 69 (coefficient = 0.06, 95 % CI=(-0.05, 0.15)) was weaker. There was limited evidence of associations between grip strength and PACC score, or that increased declines in grip strength score from midlife were associated with either of the cognitive metrics, and no interactions with sex were seen for these measures (Table 2; Fig. 2C-D).

### Associations between GBTM group classification (based on grip strength trajectory between 53 and 69) and neuroimaging and cognition at 69–71

A three-group model was chosen as the best compromise between fit, classification ambiguity, and complexity (Supplementary Data Sec. 1.3). The groups can be interpreted as a consistent below-average trend (*n* = 733; 23.8 %), above-average trend (*n* = 283; 9.2 %), and an average trend (*n* = 2062; 67.0 %; reference category). Compared to the whole NSHD sample, a slightly lower proportion of the Insight 46 participants were classified with a low trend (21.1 %; *n* = 94), and a higher proportion classified with a high trend (13.9 %; *n* = 62), with a similar proportion of reference individuals (65.0 %; *n* = 290). The trajectories and assignments for these individuals are presented in Fig. 1B. The analysis of the relationship with each of the neuroimaging and cognitive outcomes included 428 participants with complete data on covariates at age 53.

The relationship between grip strength trajectory group and outcomes was largely consistent with earlier results (Table 3; Fig. 2E-H). In particular, there was reasonably strong evidence that below-average group membership was associated with an estimated 13.38cm^3^ lower WBV at age 69–71 (95 % CI=(-24.12, -2.64)) compared to reference group individuals, adjusting for sex, body size, TIV, sociodemographics, and behavioural and health risk factors (Fig. 2E). In addition, there was some evidence of a complementary trend where women in the above-average group had higher whole-brain volume at 69–71, estimated at 18.30cm^3^ (95 % CI=(1.34, 35.26); *n* = 34 high group female participants) compared to the reference group, after adjusting for all covariates, although a similar association was not seen in men (interaction *p* = 0.08).

**Table 3:** Associations between grip strength trajectory group membership and brain health and cognitive measures at 69–71

	Group membership (vs reference, *n* = 275)		
	Low (*n* = 93)		High (*n* = 60)
***Brain health measures***			
WBV (cm^3^)	-13.38* (-24.12, -2.64)	**M:**	-3.65 (-21.92, 14.62)
		**F:**	18.30* (1.34, 35.29)
Global WMHV (mL)	0.86 (0.67, 1.11)		1.07 (0.80, 1.44)
***Cognitive measures***			
PACC (z-score)	-0.06 (-0.20, 0.09)		-0.01 (-0.18, 0.16)
Matrix reasoning (z-score)‡	-0.33† (-0.58, -0.13)		-0.08 (-0.34, 0.14)

*p < 0.05

† BCa bootstrap 95% CI does not contain 0

‡ 95% CIs obtained from BCa bootstrap with 2,000 replicates

Coefficients (95 % CIs) are derived from multivariable linear regression models or GLMs and give the estimated mean difference between the low or high group versus the reference group for WBV and the multiplicative difference between low or high group versus the reference group for WMHV. All models included 428 participants with complete baseline covariates (age 53), and were adjusted for sex, age at scan/visit, height, weight, physical activity, and vascular risk score at age 53, adult SEP, education, and a binary indicator of cognitive or neurological impairment at age 69–71. Additional adjustments were for weight squared (WMHV), TIV (brain measures), and childhood cognitive ability (cognitive measures). Values given to 2 d.p.

In line with earlier results, consistently below-average grip strength was also associated with lower non-verbal reasoning: an estimated 0.33 lower matrix reasoning score compared to the reference group (BCa bootstrapped 95 % CI=(-0.58, -0.13); Fig. 2H). However, there was no statistical evidence for a positive effect of above-average grip strength on nonverbal reasoning, nor was there any evidence of any association or trends between PACC score or WMHV and the below- or above-average trajectory groups (Fig. 2F-G). Models relating a unit increase (from 0 to 1) in posterior probability of low or high group membership to the outcomes identified similar trends (Table S3).

## Discussion

In a 446 participant sub-sample from the British 1946 birth cohort, we found converging evidence that below-average grip strength from age 53 was modestly associated with lower whole-brain volume (WBV) at age 69–71. Conversely, above-average grip strength was associated with higher WBV, which was more pronounced in females. For white matter hyperintensity volume (WMHV) the findings were less consistent. Lower grip strength from age 53 was associated with lower WMHV but above-average declines in grip strength were associated with increased WMHV. Lastly, lower grip strength was consistently associated with lower nonverbal (matrix) reasoning scores, a measure of fluid cognition. All results were independent of body size, physical activity, and sociodemographic, vascular and health risk factors, plus head size (neuroimaging measures) or childhood cognition (cognitive measures).

Consistent with our findings, previous studies have generally found that higher levels of objective physical function measures, including grip strength, are cross-sectionally associated with higher WBV [20, 47], and fluid reasoning ability [13, 48]. Consistently above-average grip strength was also associated with higher WBV in women, which may reflect sex differences in the rate of age-related cortical atrophy [49], though given that maximum grip strength at age 60–64 and 69 was positively associated with WBV independently of sex, it may be that this effect was more pronounced in women in this sample by chance (see also Supplementary Data Sec. 2.1).

Higher grip strength was weakly associated with increases in global WMHV, which is at odds with previous work [21]. That said, there was no evidence that individuals with below- or above-average grip strength from midlife differed in WMHV compared to those with average trajectories; nor any evidence of a positive association between grip strength at age 69 and WMHV. However, above-average decline in grip strength was weakly associated with higher WMHV, which is consistent with studies reporting longitudinal associations between declines in other physical function measures (e.g., gait speed, chair rises) and increased WMHV [50–52]. Studies are needed in other cohorts across a range of ages to examine this discrepancy.

Maximum grip strength is positively associated with primary motor cortex volumes and cerebellar volume in older adults [53]. Given that changes in cerebellar morphology are associated with age-related motor and cognitive decline [54], differences in WBV may reflect structural changes in regions involved in motor control and coordination [55]. Such changes may result from broader alterations, for example to neuromodulation [56], which may in turn be associated with cellular (e.g., inflammatory) pathways common to both physical and cognitive decline [57]. More work is needed to characterise these pathways and associated biomarkers, in particular to understand age and sex differences in the effect of risk factors on grip strength declines [58] and how this may relate to brain structure and cognitive ageing.

Furthermore, while we lack the baseline imaging metrics to confirm this, lower WBV and higher WMHV may indicate an increased rate of brain atrophy and accelerated white matter lesion accumulation, respectively, given that both occur in healthy ageing [15]. Increased global WMHV and whole-brain atrophy are known markers of cerebral small vessel disease [14, 59], which in turn is predictive of dementia, stroke, and mortality [18]. Future work should monitor the cohort to assess the extent to which associations between indicators of physical function and markers of brain health relate to clinical outcomes such as stroke and dementia.

This study has several strengths, including the large sample of narrow age range, and measures of grip strength up to 17 year prior to cognitive tests and sensitive neuroimaging metrics. We were also able to control for numerous life-course covariates in all models, and analyses with cognitive outcomes additionally adjusted for childhood cognition, which is notable since childhood cognitive ability is highly predictive of scores decades later [32]. In addition, we used a statistical model to identify groups of individuals with similar grip strength trajectories, rather than the subjective methods that have been used previously to classify objective physical function in this cohort [42, 60].

There are also several limitations to the present work. Firstly, though the NSHD is representative of the British-born population at the time, it is ethnically homogeneous. Secondly, the Insight 46 subsample have a higher SEP, are more educated, active, and generally healthier than the main NSHD sample. This may mean associations in the subsample are underestimates [24]. Though we were able to partially account for this by fitting the GBTM on the whole NSHD sample (*n* = 3078), this method has associated limitations (Supplementary Data Sec. 1.4), and should be considered a tool to describe patterns of trends rather than a means to identify distinct developmental trajectories [44]. Lastly, while we were able to adjust for self-reported physical activity in all models, this measure is unlikely to completely capture variation in cardiorespiratory fitness.

## Conclusions

Taken together, we have shown in a sample of individuals from a British birth cohort that below- or above-average trends in maximum grip strength from midlife are relatively consistent predictors of later-life whole-brain volumes and nonverbal reasoning, and that above-average decline in grip strength may also modestly predict increased global white-matter hyperintensity volumes. These results provide preliminary evidence for the potential utility of grip strength in monitoring cognitive and neurological function, as suggested by several recent reviews [4–6]. Given that tests of grip strength are quick, non-invasive, and relatively inexpensive, one could envision their future use in primary care, in combination with normative population measures [61], to provide insights into brain structure [6], and to help assess the risks of associated clinical manifestations such as stroke and dementia [20]. We suggest that current and future longitudinal cohort studies include measures of objective physical function from an earlier age, to better understand their temporal dynamics in younger cohorts, to assess whether there exist groups of individuals following distinct developmental trajectories, and to investigate to what extent such trajectories relate to structural and functional changes in the brain.

## Supplementary Information

**Additional file 1.** Inclusion flow-chart (Figure S1), additional information on group-based trajectory modelling (GBTM) including rationale (Sec. 1.2), model selection (Sec. 1.3; Table S1-S2), and limitations (Sec. 1.4), plus additional data (Sec. 2) from models relating increases in posterior probability of membership of the low or high trajectory group to each of the outcomes (Table S3), and models relating group assignment from a GBTM fitted only on included participants (Figure S2) to the outcomes (Table S4).

## Data Availability

Data used in this publication (10.5522/NSHD/Q101; 10.5522/NSHD/Q102; 10.5522/NSHD/Q103) are available to bona fide researchers upon request to the NSHD Data Sharing Committee via a standard application procedure. Further details can be found at http://www.nshd.mrc.ac.uk/data.

## Abbreviations

BCa: Bias-Corrected and accelerated
BIC: Bayesian Information Criterion
BMI: Body Mass Index
CI: Confidence Interval
CVD: Cardiovascular Disease
DSST: Digit Symbol Substitution Test
FHS: Framingham Heart Study
FNAME-12: 12-item Face-Name test
GBTM: Group-Based Trajectory Modelling
GLM: Generalised Linear Model
MRC: Medical Research Council
MRI: Magnetic Resonance Imaging
MMSE: Mini-Mental State Exam
NSHD: National Survey of Health and Development
PACC: Preclinical Alzheimer Cognitive Composite
PET: Positron Emission Tomography
SD: Standard Deviation
SE: Standard Error
SEP: Socio-Economic Position
SPM: Statistical Parametric Mapping
TIV: Total Intracranial Volume
WASI: Wechsler Abbreviated Scale of Intelligence
WBV: Whole-Brain Volume
WMHV: White Matter Hyperintensity Volume
