# Correction to: Grip strength from midlife as an indicator of later-life brain health and cognition: evidence from a British birth cohort

**DOI:** 10.1186/s12877-021-02445-x

**Published:** 2021-09-29

**Authors:** Quentin Dercon, Jennifer M. Nicholas, Sarah-Naomi James, Jonathan M. Schott, Marcus Richards

**Affiliations:** 1 MRC Cognition and Brain Sciences Unit, University of Cambridge, Cambridge, United Kingdom; 2 Department of Medical Statistics, London School of Hygiene & Tropical Medicine, London, United Kingdom; 3 MRC Unit for Lifelong Health and Ageing at UCL, University College London, London, United Kingdom; 4 Dementia Research Centre, UCL Queen Square Institute of Neurology, University College London, London, United Kingdom

**Correction to: BMC Geriatr 21, 475 (2021)**

**https://doi.org/10.1186/s12877-021-02411-7**

After publication of this article [1], the authors noticed 3 empty cells in Table 1, whereas they should read “< 0.001”.

The original article [1] has been updated.
