# Supplementary material for: Grip strength from midlife as an indicator of later-life brain health and cognition: evidence from a British birth cohort
Source: BMC Geriatr. 2021 Aug 31;21:475. doi: 10.1186/s12877-021-02411-7 (PMC8406895; doi:10.1186/s12877-021-02411-7)
Supplement: Supplementary file 1 — Additional file 1. Inclusion flow-chart (Figure S1), additional information on group-based trajectory modelling (GBTM) including rationale (Sec. 1.2), model selection (Sec. 1.3; Table S1-S2), and limitations (Sec. 1.4), plus additional data (Sec. 2) from models relating increases in posterior probability of membership of the low or high trajectory group to each of the outcomes (Table S3), and models relating group assignment from a GBTM fitted only on included participants (Figure S2) to the outcomes (Table S4). [file 12877_2021_2411_MOESM1_ESM.pdf]

Supplementary Data

1. Methods

1.1 Participant inclusion

Figure S1: Inclusion flow-chart for the present study.

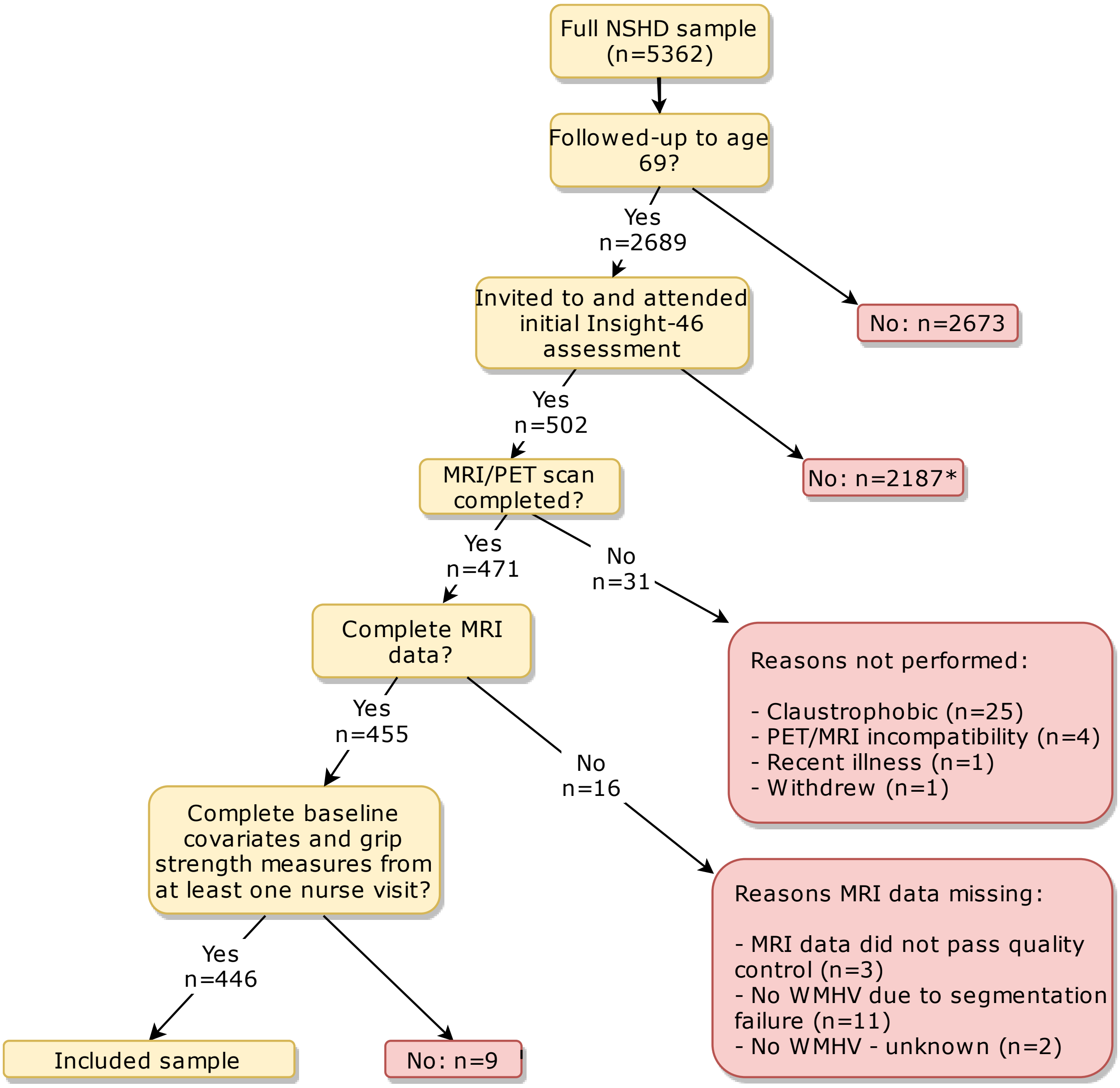

More information on recruitment procedures relevant to the Insight 46 sub-study (*) can be found elsewhere [1–3].

1.2 Group-based trajectory modelling: rationale

Though previous work on this sample has used *a priori* classification to relate trajectories of change in physical function to behavioural and health outcomes [4], this requires group membership criteria to be arbitrarily defined. GBTM was used here to extend this work, using a data-driven model to identify the most likely set of grip strength z-score trajectory groups present in our sample, before subsequently relating group membership to the distal cognitive and neuroimaging outcomes.

A longitudinal extension of finite mixture modelling, GBTM is a semi-parametric method that identifies latent groups of individuals based on their patterns of responses, and models each group with their own probability distribution function [5–7]. Given the continuous nature of the outcome (grip strength z-score), normal distributions were assumed for all groups, with a quadratic form assumed for the independent variable, age at the nurse visit (higher-order polynomial forms are not possible with only three time points). Bootstrapping with 500 replicates was used to calculate the standard error for the coefficients for age at the nurse visit and quadratic term for age at the nurse visit. For parsimony, quadratic terms with weak statistical evidence for inclusion were dropped (Normal-approximation 95% confidence interval including the null value of 0), but all linear terms were retained, in line with recommendations [8].

To find the optimal number of groups, models with increasing numbers were fitted using the *traj* package in Stata [9] and posterior probabilities of group membership calculated for each group for each individual. The maximum of these posterior probabilities was then used to classify each individual into their most likely group (maximum probability assignment rule) [6]. Models were then compared using three metrics: the Bayesian Information Criterion (BIC); the proportion of the sample classified in each group (so that one group is not overly large or small); and the mean posterior probabilities of those classified into each group (with higher values indicating lower classification ambiguity [10]). In addition to group assignment, the posterior probabilities for each individual were also used as a continuous measure and related separately to each of the distal cognitive and brain health outcomes. Lastly, model selection and distal outcome analyses were repeated using measures from the included Insight 46 subsample only, in order to compare trends in the identified trajectory groups to those found in the whole NSHD cohort.

1.3 Group-based trajectory modelling: model selection

As GBTM can take into account missing data (assumed missing at random [11]), all individuals in the whole NSHD sample with at least one grip strength measure were included in the modelling (n=3078), for a total of 7019 observations (3 influential outlier measures at age 53 were excluded). According to the BIC alone, there was strong evidence that a 4-group model represented the best trade-off between complexity and fit (Table S1). However, while this model had reasonable average posterior probabilities given classification (mean of all groups = 0.74), it contained two fairly small groups (4.4% and 4.6% of the population) representing the highest and lowest trends, and two larger groups (>40% of population each).

The 3-group model, meanwhile, had an only slightly more negative BIC than the 4-group model (-9494.2 versus -9483.0) but had higher average posterior probabilities given classification and comprised three reasonably large groups representing a low, average, and high trend over time. Since the smallest group should ideally contain at least 5% of the population [8], and posterior probabilities

given classification should be close to or >0.8 for all groups to consider classification to be fairly unambiguous [10], the 3-group model was ultimately chosen as the best compromise between complexity and ability to provide a clear classification of grip strength trajectories. There was no statistical evidence of a quadratic trend in any of the three groups, so this term was omitted. Evidence of a linear trend was similarly limited (Normal-approximation $p>0.13$ for all groups), but as noted above this term was retained in all three groups.

Table S1: BIC, average posterior probability (range), and smallest group size (%), for the models considered.

No. groups	BIC (*n*=3078)	Mean posterior probabilities given membership (range)	Minimum group membership (%)
1	-9911.1	-	-
2	-9574.4	0.83 (0.81-0.85)	37.8
3	-9494.2	0.78 (0.78-0.79)	9.2
4	-9483.0	0.74 (0.72-0.79)	4.4
5	-9493.7	0.73 (0.70-0.79)	0.3

Model selection was then repeated using grip strength measures from the included Insight 46 participants only (1338 total observations from *n*=446 individual participants). Unlike for the models fit on the whole NSHD cohort, there was little evidence from the BIC to suggest that the 3, 4, or 5-group model represented a better trade-off between complexity and fit (Table S2).

Table S2: BIC, average posterior probability (range), and smallest group size (%), for the models considered in the included subsample (*n*=446).

No. groups	BIC (*n*=446)	Mean posterior probabilities given membership (range)	Minimum group membership (%)
1	-1839.01	-	-
2	-1757.20	0.88 (0.87-0.88)	47.3
3	-1741.46	0.84 (0.81-0.86)	4.9
4	-1738.10	0.83 (0.79-0.90)	1.6
5	-1738.47	0.84 (0.80-0.91)	1.6
6	-1745.09	0.78 (0.71-0.88)	1.8

Average posterior probabilities given membership were also similar between the three models. However, both the 4- and 5-group models contained at least one very small group of 7 individuals (1.6% of the population), while the smallest group in the 3-group model contained just under 5% of the population. For this reason, plus ease of comparison with the whole NSHD trajectory groups, the

3-group model was chosen. As before, there was no statistical evidence of a quadratic trend in any of the groups, so these terms were dropped.

1.4 Group-based trajectory modelling: limitations

It should be noted the GBTM approach has several limitations and should be considered exploratory. Firstly, simulation studies have shown that having a non-natural starting point (i.e., age 53) and limited numbers of longitudinal observations (3 in our case) are likely to affect the validity of class enumeration [10]. Though our choice of a GBTM over more complex growth mixture models (GMM) [12, 13] was in part to reduce overfitting in the face of these issues, GBTMs assume that residual variance is fixed across classes and time-points, and hence that individuals within each group are homogeneous. As this assumption is unlikely to hold, it means that identified groups should be seen as approximations rather than literally existing [11]. Indeed, given the groups differed significantly only in intercept, it may also be that the observed standardised grip strength trajectories could equally be explained by a single mean trend with individual-specific random variation (i.e., a growth curve model). Lastly, our "classify-analyse" approach to relate group membership to the distal outcomes is known to introduce noise and systematically bias estimates downwards [14], as assignment is assumed observed in the final stage, and hence uncertainty in assignment is not considered [15].

2. Results

2.1 Associations between posterior probabilities of GBTM group membership given classification and brain health and cognition at 69-71

Models relating posterior probability of low or high group membership to each of the neuroimaging and cognitive outcomes in the 428 participants with complete covariates at age 53 (Table S3) indicated largely similar trends to models relating the outcomes to categorical group assignment. Greater posterior probability of membership of the low trajectory group was associated with lower WBV and matrix reasoning z-scores; following adjustment for sex, body size and total intracranial volume, sociodemographics, and behavioural and health risk factors, a unit increase (from 0 to 1) in posterior probability of membership of the low trajectory group was associated with an estimated 19.66cm^3 lower WBV (95% CI=(-33.29, -6.03)) and a 0.38 lower matrix reasoning z-score (BCa bootstrapped 95% CI=(-0.69, -0.11)) at age 69-71. There was little evidence of associations between posterior probability of membership of either group and WMH or PACC z-score (Table S3).

Although evidence of a sex interaction had been found in analysis of the association between membership of the high grip strength trajectory groups and WBV, this was not apparent in the analysis of posterior probability of high group membership (sex interaction p=0.12). Instead, there was a trend suggesting a 0 to 1 increase in posterior probability of membership of the high trajectory group was associated with an estimated 13.51cm^3 higher WBV at age 69-71 in both sexes, again adjusting for all

covariates (95% CI: (-2.15, 29.17)). This suggests some uncertainty as to whether the possible protective effect of high grip strength trajectory on WBV is restricted to females or may be present for both sexes.

Table S3: Associations between posterior probability of trajectory group membership and brain health and cognitive measures at 69-71

	Posterior probability of group membership	
	Low (*n*=428)	High (*n*=428)
Brain health measures		
WBV (cm^3)	-19.66** (-33.29, -6.03)	13.51 (-2.15, 29.17)
Global WMHV (mL)	0.82 (0.60, 1.13)	1.16 (0.80, 1.70)
Cognitive measures		
PACC (z-score)	-0.02 (-0.21, 0.17)	-0.02 (-0.24, 0.19)
Matrix reasoning (z-score)‡	-0.38† (-0.69, -0.11)	0.10 (-0.17, 0.36)

** $p<0.01$
† BCa bootstrap 95% CI does not contain 0
‡ 95% CIs obtained from BCa bootstrap with 2,000 replicates

Coefficients (95% CIs) are derived from multivariable linear regression models or GLMs and are interpreted as the expected difference in outcome between a participant with a posterior probability of 1 estimated from the GBTM for a given group, compared to a participant with an estimated posterior probability of 0 for that group, conditional on assignment (posterior probabilities were estimated for all 428 individuals with complete covariates at age 53 for all 3 groups). For WBV and cognitive measures the coefficient gives the difference in mean of the outcome and for WMHV this is the multiplicative effect. All models adjusted for sex, age at scan/visit, height, weight, physical activity, and vascular risk score at 53, adult SEP, education, and a binary indicator of cognitive or neurological impairment at 69-71. Additional adjustments were for TIV (brain measures), and childhood cognitive ability (cognitive measures). Values given to 2 d.p.

2.2 Comparison between trajectory groups identified in the whole NSHD cohort, and the included Insight 46 subsample

A three-group GBTM fitted on only the included 446 participants identified similar trends to that fitted on the whole NSHD cohort, with participants assigned to groups following consistent below- and above-average trajectories, and a reference group. However, the estimated mean trajectories for each of the identified groups were higher than in the whole-cohort model (Figure S2). In line with this, fewer participants were assigned to the above-average group (*n*=22) and the reference group (*n*=272), and more participants were assigned to the below-average group (*n*=152), compared to the GBTM fitted on measures from the whole NSHD cohort (*n*=62, *n*=290, and *n*=94 respectively).

Figure S2: Comparison of grip strength z-score trajectory group assignment based on the whole NSHD and the included subsample.

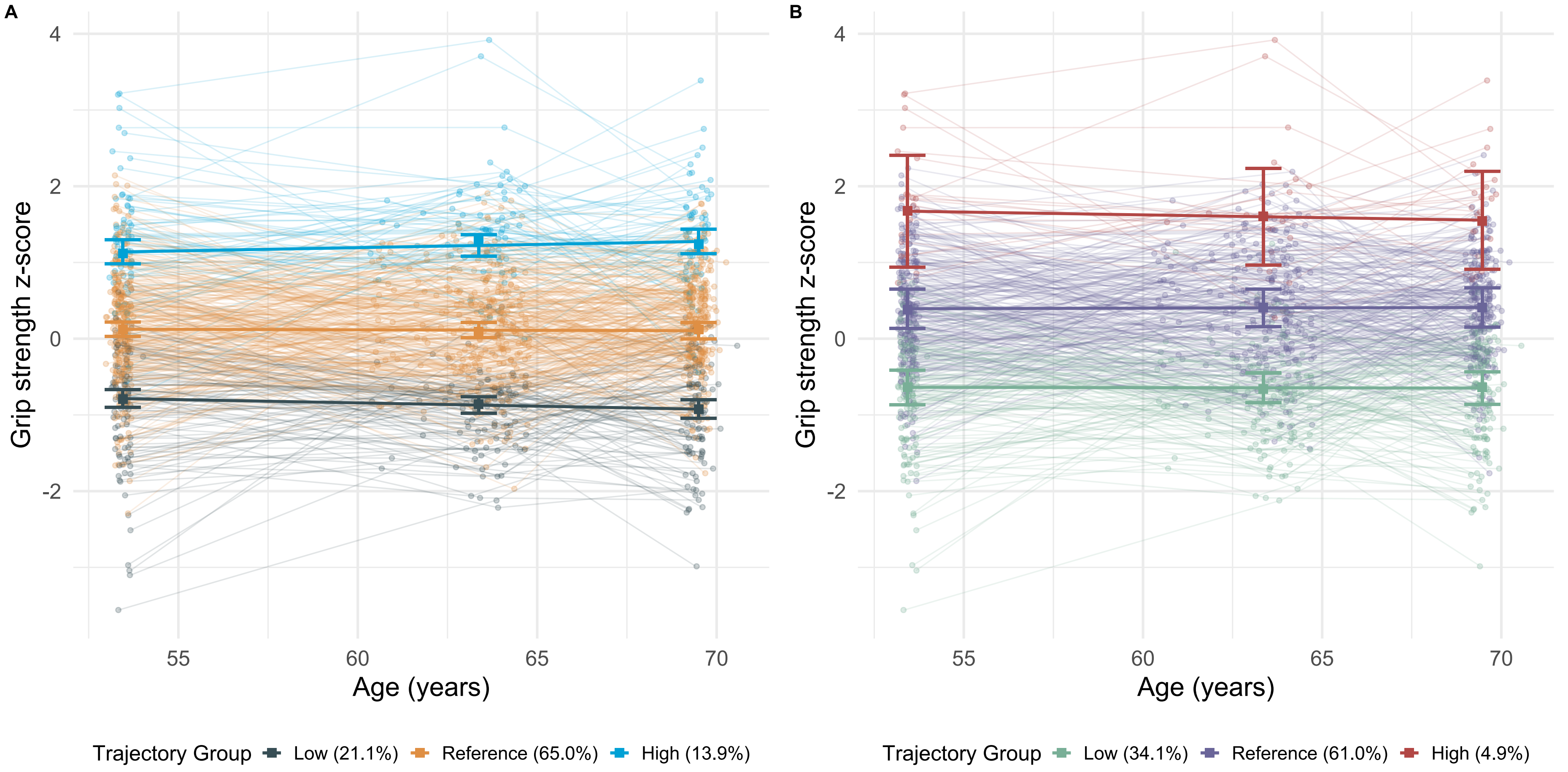

Grip strength z-score trajectories for all included Insight 46 participants (*n*=446), coloured by their assigned trajectory group based on a 3-group GBTM fitted on the whole NSHD cohort with at least one grip strength measure (*n*=3078) [**A**] and the included Insight 46 subsample (*n*=446) [**B**]. Bold lines correspond to the estimated trajectory for each of the groups derived from the GBTM, with error bars showing 95% CIs at the mean age of each study wave, and the bold squares are the mean grip strength z-score for each group at each study wave.

Despite differences in the size and nature of the identified groups when fitting the GBTM only on the included subsample, membership of the below-average trajectory group was similarly associated with lower WBV and matrix reasoning scores, compared to reference individuals (Table S4). There was little evidence of associations between group assignment and any of the other cognitive or brain health outcomes, or of any protective trends in those identified as having an above-average grip strength trajectory, though it should be noted that this group was relatively small (*n*=22).

Table S4: Comparison of associations between grip strength trajectory group and brain health and cognitive outcomes

	Group membership, based on whole NSHD (vs reference, *n*=275)		Group membership, based on included subsample (vs reference, *n*=258)	
	Low (*n*=93)	High (*n*=60)	Low (*n*=148)	High (*n*=22)
Brain health measures				
WBV (cm^3)	-13.38*<br>(-24.12, -2.64)	**M:** -3.65<br>(-21.92, 14.62)<br>**F:** 18.30*<br>(1.34, 35.29)	-11.96*<br>(-21.28, -2.64)	1.55<br>(-18.12, 21.23)
Global WMHV (mL)	0.86<br>(0.67, 1.11)	1.07<br>(0.80, 1.44)	0.92<br>(0.74, 1.15)	1.07<br>(0.67, 1.69)
Cognitive measures				
PACC (z-score)	-0.06<br>(-0.20, 0.09)	-0.01<br>(-0.18, 0.16)	0.01<br>(-0.11, 0.14)	-0.10<br>(-0.37, 0.17)
Matrix reasoning (z-score)‡	-0.33†<br>(-0.58, -0.13)	-0.08<br>(-0.34, 0.14)	-0.20†<br>(-0.39, -0.02)	0.15<br>(-0.10, 0.35)

* p<0.05
† BCa bootstrap 95% CI does not contain 0
‡ 95% CIs obtained from BCa bootstrap with 2,000 replicates

Group membership refers to the trajectory group assignment based on 3-group GBTMs fit on either the whole NSHD (*n*=3078) or the included Insight 46 subsample (*n*=446). Coefficients (95% CIs) are derived from multivariable linear regression models or GLMs and give the estimated mean difference between the low or high group versus the reference group for WBV and the multiplicative difference between low or high group versus the reference group for WMHV. All models included 428 participants with complete baseline covariates (age 53), and were adjusted for sex, age at scan/visit, height, weight, physical activity, and vascular risk score at age 53, adult SEP, education, and a binary indicator of cognitive or neurological impairment at age 69-71. Additional adjustments were for weight squared (WMHV), TIV (brain measures), and childhood cognitive ability (cognitive measures). Values given to 2 d.p.

3. References

1. Lane CA, Barnes J, Nicholas JM, Sudre CH, Cash DM, Parker TD, et al. Associations between blood pressure across adulthood and late-life brain structure and pathology in the neuroscience substudy of the 1946 British birth cohort (Insight 46): an epidemiological study. Lancet Neurol. 2019;18:942–52.

2. Lu K, Nicholas JM, Collins JD, James SN, Parker TD, Lane CA, et al. Cognition at age 70: Life course predictors and associations with brain pathologies. Neurology. 2019;93:E2144–56.

3. James SN, Lane CA, Parker TD, Lu K, Collins JD, Murray-Smith H, et al. Using a birth cohort to study brain health and preclinical dementia: Recruitment and participation rates in Insight 46. BMC Res Notes. 2018;11. doi:10.1186/s13104-018-3995-0.

4. Cooper R, Muniz-Terrera G, Kuh D. Associations of behavioural risk factors and health status with changes in physical capability over 10 years of follow-up: The MRC National Survey of Health and Development. BMJ Open. 2016;6:e009962.

5. Nagin DS, Land KE. Age, criminal careers, and population heterogeneity: Specification and estimation of a nonparametric, mixed Poisson model. Criminology. 1993;31:327–62. doi:10.1111/j.1745-9125.1993.tb01133.x.

6. Nagin DS. Analyzing developmental trajectories: A semiparametric, group-based approach. Psychol Methods. 1999;4:139–57. doi:10.1037/1082-989X.4.2.139.

7. Nagin DS. Group-Based Modeling of Development. Cambridge, Mass.: Harvard University Press; 2005.

8. Andruff H, Carraro N, Thompson A, Gaudreau P, Louvet B. Latent Class Growth Modelling: A Tutorial. Tutor Quant Methods Psychol. 2009;5:11–24.

9. Jones BL, Nagin DS. A Note on a Stata Plugin for Estimating Group-based Trajectory Models. Sociol Methods Res. 2013;42:608–13.

10. van der Nest G, Lima Passos V, Candel MJJM, van Breukelen GJP. An overview of mixture modelling for latent evolutions in longitudinal data: Modelling approaches, fit statistics and software. Adv Life Course Res. 2020;43:100323.

11. Nagin DS, Odgers CL. Group-based trajectory modeling in clinical research. Annu Rev Clin Psychol. 2010;6:109–38. doi:10.1146/annurev.clinpsy.121208.131413.

12. Muthén B, Shedden K. Finite mixture modeling with mixture outcomes using the EM algorithm. Biometrics. 1999;55:463–9. doi:10.1111/j.0006-341X.1999.00463.x.

13. Muthén BO, Muthén LK. Integrating person-centered and variable-centered analyses: Growth mixture modeling with latent trajectory classes. Alcohol Clin Exp Res. 2000;24:882–91.

14. Bolck A, Croon M, Hagenaars J. Estimating latent structure models with categorical variables: One-step versus three-step estimators. Polit Anal. 2004;12:3–27. doi:10.1093/pan/mph001.

15. Bray BC, Lanza ST, Tan X. Eliminating Bias in Classify-Analyze Approaches for Latent Class Analysis. Struct Equ Model. 2015;22:1–11. doi:10.1080/10705511.2014.935265.
